# IoT empowered smart cybersecurity framework for intrusion detection in internet of drones

**DOI:** 10.1038/s41598-023-45065-8

**Published:** 2023-10-27

**Authors:** Syeda Nazia Ashraf, Selvakumar Manickam, Syed Saood Zia, Abdul Ahad Abro, Muath Obaidat, Mueen Uddin, Maha Abdelhaq, Raed Alsaqour

**Affiliations:** 1 Department of Computer Science, Sindh Madressutal Islam University, Karachi, Pakistan; 2 National Advanced IPv6 Centre (NAv6), Universiti Sains Malaysia, 11800 Gelugor, Penang Malaysia; 3 Software Engineering Department, Sir Syed University of Engineering and Technology, Karachi, Pakistan; 4 Department of Computer Science, Faculty of Engineering Science and Technology, İqra University, Karachi, Pakistan; 5 Department of Computer Science, City University New York, New York, NY 10036 USA; 6 College of Computing and Information Technology, University of Doha for Science and Technology, 24449 Doha, Qatar; 7 Department of Information Technology, College of Computer and Information Sciences, Princess Nourah Bint Abdulrahman University, P.O. Box 84428, Riyadh, 11671 Saudi Arabia; 8 Department of Information Technology, College of Computing and Informatics, Saudi Electronic University, Riyadh, 93499 Saudi Arabia

**Keywords:** Engineering, Mathematics and computing

## Abstract

The emergence of drone-based innovative cyber security solutions integrated with the Internet of Things (IoT) has revolutionized navigational technologies with robust data communication services across multiple platforms. This advancement leverages machine learning and deep learning methods for future progress. In recent years, there has been a significant increase in the utilization of IoT-enabled drone data management technology. Industries ranging from industrial applications to agricultural advancements, as well as the implementation of smart cities for intelligent and efficient monitoring. However, these latest trends and drone-enabled IoT technology developments have also opened doors to malicious exploitation of existing IoT infrastructures. This raises concerns regarding the vulnerability of drone networks and security risks due to inherent design flaws and the lack of cybersecurity solutions and standards. The main objective of this study is to examine the latest privacy and security challenges impacting the network of drones (NoD). The research underscores the significance of establishing a secure and fortified drone network to mitigate interception and intrusion risks. The proposed system effectively detects cyber-attacks in drone networks by leveraging deep learning and machine learning techniques. Furthermore, the model's performance was evaluated using well-known drones’ CICIDS2017, and KDDCup 99 datasets. We have tested the multiple hyperparameter parameters for optimal performance and classify data instances and maximum efficacy in the NoD framework. The model achieved exceptional efficiency and robustness in NoD, specifically while applying B-LSTM and LSTM. The system attains precision values of 89.10% and 90.16%, accuracy rates up to 91.00–91.36%, recall values of 81.13% and 90.11%, and F-measure values of 88.11% and 90.19% for the respective evaluation metrics.

## Introduction

**Figure 1:**
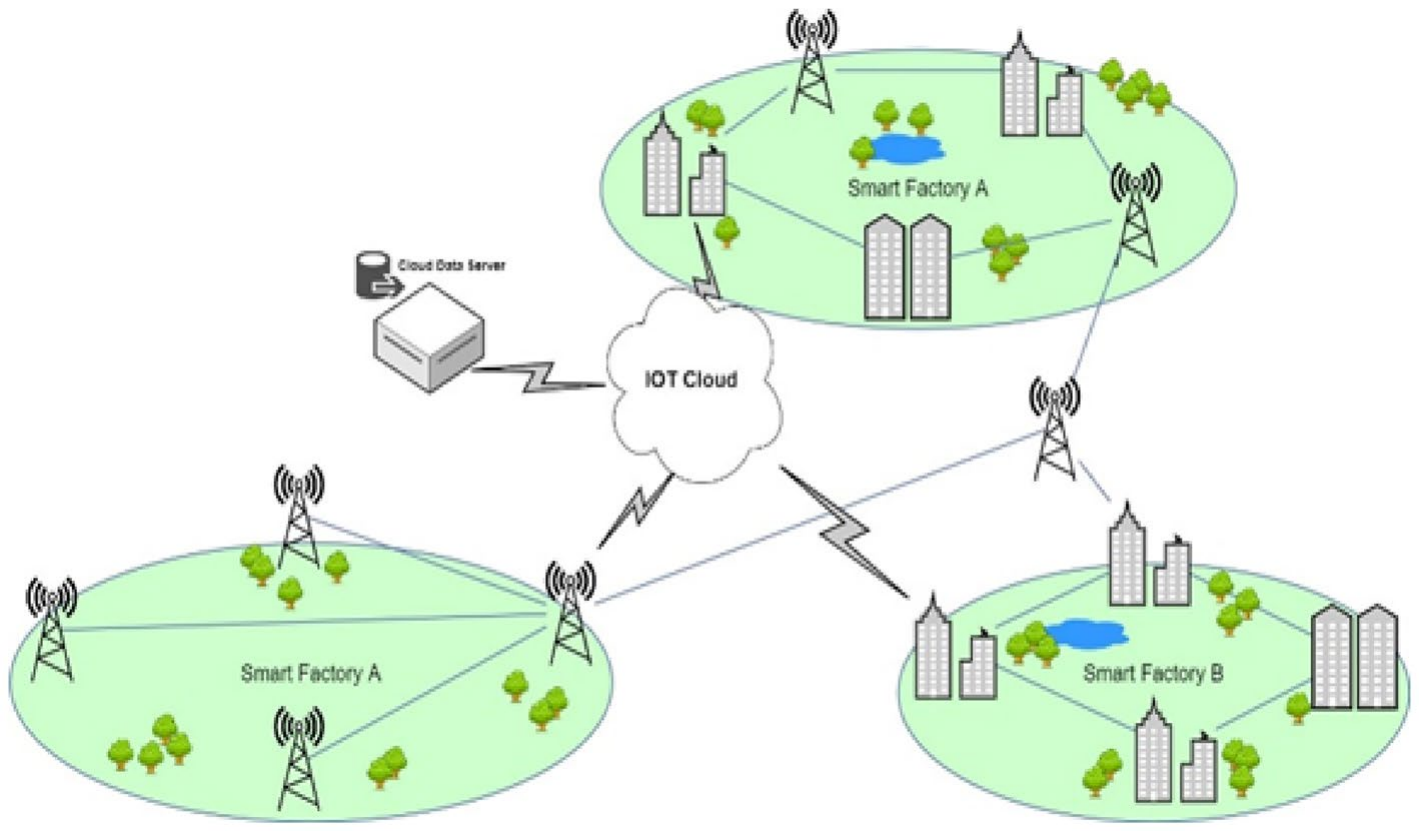
The Current Infrastructure of Smart Factories.

The growing popularity of the Internet of Drones (IoD) is ascribed to the ongoing downsizing of sensors and chipsets and pervasive wireless communication. Tiny miniature drones, such as micro drones and quadcopters, have proliferated due to advancements in robot technology and unmanned aerial vehicles^1–3^. The ability of these miniature drones to instantly penetrate any monitoring system to track physical objects is a significant advantage. It is used in a variety of fields, including disaster response, industrial surveillance, military applications, search and rescue, precision agriculture, delivery, and shipping. Unmanned aerial vehicles (UAVs), often known as drones, are aerial aircraft without human pilots. They are helpful for various things, including weather forecasting and aerial photography. Aerodynamics forces frequently use UAVs to provide them access to remote machine control capabilities^4–6^. The aspects of diverse businesses have been influenced by analogous commercial applications, which affect everyone. UAVs are helpful tools for assessment and control since they can often capture aerial data and easily communicate it to the base station. The expanding use of drone technology has raised concerns about liability, privacy, regulation, and security^1,4–6^. Drone technology has become widespread since reduced-sized UAVs have so many benefits for privacy, distribution, and shipping^7^. However, these drones have significant privacy and security concerns, such as Intrusion Detection Systems (IDS). The integration of the Internet of Things (IoT) and wireless sensors that might be used in miniature drones has been made to conceive smart drones have been a growing area of research in recent years. Various technologies, including sensors, transmitters, and cameras, can enhance the functionality and effectiveness of drones in a wide range of complex applications. Small drones are providing new opportunities for the defense and public sectors. Tiny drones are susceptible to security and privacy problems because of inadequate design. The Internet of Drones (IoD) is a derivative of the Internet of Things in which drones are linked via Internet technology and offer new routes while presenting security and privacy challenges^6^. Changing IoDs is the fundamental architecture, and design is essential to improve security and dependability. Traditionally, the structure of a conventional drone was constructed using a layered design, as shown in Fig. 1.

In a typical compact structure for commercial drones, a copter is integrated with a camera as the first module, called the drone module. Smart drones are linked to terrestrial networks using IoT gateways^8^. In this scenario, communication is provided by IoT gateways, like a terrestrial station's network that is cloud-equipped. After receiving the data from the IoT hub, the computational module examines the data stream. The outcomes of the computational analysis are entered into the database at the storage module and then sent to the visualization module for further examination.

On the other hand, one disadvantage is that it does not have the functionality to provide privacy and data security. IoT and drones are combined in the modern idea of the Internetwork of Drone Things (IDT), which enables drones to connect to a device that contains an IoT network. The current study proposes the IDT concept as a solution to security and privacy challenges. The main concerns for the IoD's implementation are the Problem Definition of IDT security. Numerous prospects might be investigated for the secure implementation of the IoD with the advancements in the ML field. The current research solves the issue by utilizing ML approaches to complete the IoD network.

The work that is being presented shows how a data-analysis-based smart drone capacity has been created by enhancing IoT and drones. Meanwhile, Blockchain technology is used in smart drones to provide security and privacy. The seven components of the suggested architecture are the modules of edge computational, drone, transmission, storage, security, processing, and Sensors for visualization 2022, 22, 2630 3 of 25.

**Figure 2:**
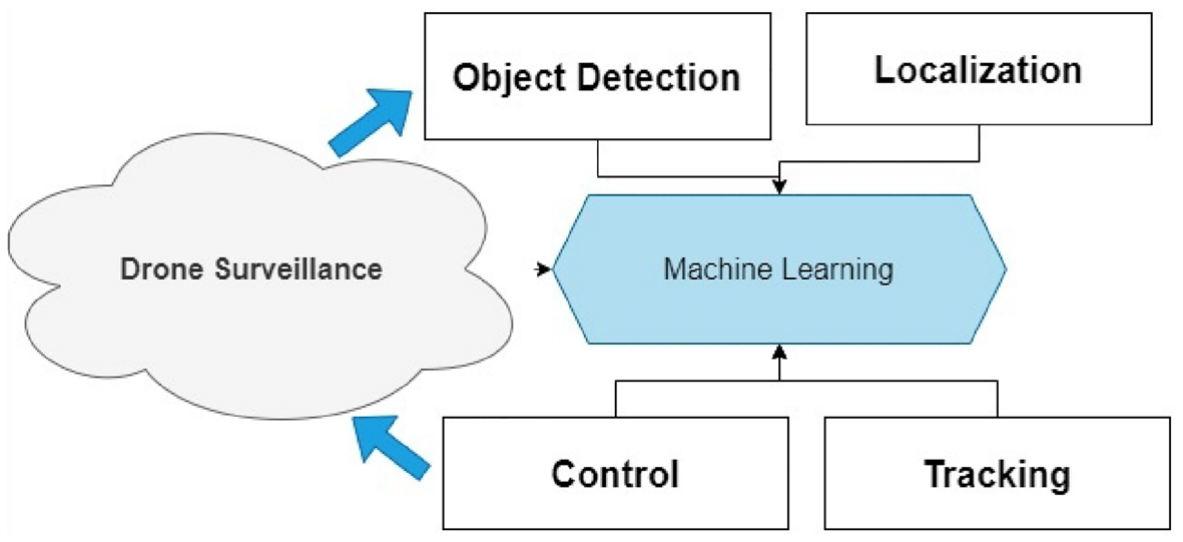
Smart Framework Layered Architecture of Drone Attacks.

However, there are numerous approaches for enabling cognitive tasks in Dragnet. For instance, the main Dragnet enabling technologies are detection, localization, tracking, and control, as shown in Fig. 2. We give an overview of each of the main enabling approaches in this section, as well as the following technological challenges and unresolved issues. Dragnet, in general, creates an intelligent amateur drone surveillance system by acting as a transparent link between the social world (social behavior, human demand, etc.) and the physical world (with available real/imaginary items, amateur drones, birds, and authorized drones). Dragnet is based on a synthetic technique of understanding learning, and it consists of four basic essential cognitive activities that must be accomplished in the following order: (1) Detecting objects, (2) Analyzing data, (3) Knowledge (Discovery and Semantic), and (4) Smart decision marking.

The most fundamental cognitive function in Dragnet is detection, which takes information from social networks and the physical environment using various active and passive surveillance technologies (such as sensors, cameras, crowds of people, or radars). Data analytics is a fundamental cognitive process that uses different surveillance data to locate and track amateur drones, identify intruders, and detect intrusions. The cognitive goal of semantic derivation and knowledge discovery attempts to enable the objects in Dragnet to automatically derive the semantics from examined data and make them self-aware and understandable. Additionally, specific usage patterns or rules can be found as knowledge based on the studied data and semantics, which requires objects in Dragnet to be intelligent. Finally, decision-making is a fundamental cognitive activity that determines broad decisions concerning the existence of amateur drones and actions to manage them (e.g., jamming, destroying, or capturing).

### Enhancing security in IoT drones: factors for smart cybersecurity implementation

Smart cybersecurity for IoT drones involves specific considerations and features to ensure the secure operation of these interconnected devices. Here are some factors that make cybersecurity smart for IoT drones:

#### Encryption and secure communication

Smart cybersecurity for IoT drones involves using robust encryption algorithms to protect data transmission between the drones and the base station or other connected devices. Secure communication protocols, such as Transport Layer Security (TLS), are implemented to safeguard data integrity and prevent unauthorized access.

#### Authentication and access control

Thanks to smart cybersecurity, only authorized parties may access and control the drones. Strong authentication mechanisms are used to confirm the identity of users and devices, such as multi-factor authentication, digital certificates, or biometric verification. Access control measures restrict privileges and permissions based on user roles and responsibilities.

#### Firmware and software security

IoT drones are powered by software and firmware. Implementing secure coding techniques, performing frequent security upgrades and patches, and choosing reputable software sources to reduce vulnerabilities are all part of smart cybersecurity. Secure boot techniques and code integrity checks help guard against unauthorized firmware alterations and guarantee its legitimacy.

#### Intrusion detection and prevention

Intrusion Detection and Prevention Systems (IDPS) are included in smart cybersecurity for IoT drones to monitor network traffic, spot irregularities, and recognize potential cyber threats. These systems use anomaly detection methods and machine learning algorithms to spot malicious activity and take preventative steps to reduce risks.

#### Threat intelligence and analytics

To identify new threats, patterns, and trends, smart cybersecurity uses threat intelligence feeds, data analytics, and machine learning algorithms. Real-time analysis of drone data and network traffic helps identify potential vulnerabilities and enables proactive defense mechanisms.

#### Physical security measures

Cybersecurity for IoT drones extends beyond digital protection. Smart cybersecurity ensures physical security measures, such as tamper-resistant enclosures, anti-tampering mechanisms, and geofencing, to prevent unauthorized physical access to drones and protect them from theft or sabotage.

#### Security monitoring and incident response

Smart cybersecurity continuously monitors drone systems, network traffic, and data flows. Security Operation Centers (SoCs) equipped with advanced monitoring tools and automated alert systems enable real-time threat detection and incident response. Incident response plans are in place to quickly mitigate and recover from security incidents.

#### Privacy and data protection

Smart cybersecurity for IoT drones emphasizes privacy and data protection. Personal and sensitive data collected by drones are handled following privacy regulations. Encryption, anonymization, and data minimization techniques protect privacy rights and ensure compliance with privacy laws.

Considering these aspects, smart cybersecurity for IoT drones provides a robust defense against cyber threats, protects data privacy, and ensures these interconnected devices' safe and secure operation. The focus of this paper falls in the category of IDS, such as anomaly-based IDS, which is more specifically on the Deep Learning (DL) mechanism. DL approaches performed better than machine learning (ML) algorithms due to learning and training modules^8^. Thus, this can provide a cost-effective and efficient IDS that is highly mandatory to keep the network security of Drones.

The main contributions of this paper are discussed as follows:

- The modular structure of an IoT-enabled drone framework is introduced to ensure robust security and privacy within the drone network.
- The hybrid ML and DL techniques enforce stringent security measures during data transmission from drones to the base station.
- The proposed framework presents intercommunication network properties where networking data, IoT sensors, and drone data are managed securely.
- Benchmark datasets are employed to test the efficacy of the proposed framework rigorously. The performance evaluation metrics employed in the results and discussion section include precision, recall, F1-score, and classification accuracy.

On the other hand, another aspect that needs more consideration is the data collection hierarchy. Till now, no standardized process has been presented that shows the working operation of drone-enabled data collection, organization, management, and optimization. This type of problem poses a severe problem in the IoD environment's future development. To tackle this scenario, this work highlights a technological integration prospect, such as the role of Artificial Intelligence (AI) in the Internet of Drones (IoD). The mentioned technological improvement helps in the designing of a standardized hierarchy, such as:

(i) drone-enabled data capture,
(ii) separation and filtering,
(iii) examination,
(iv) illustration,
(v) storage logs,
(vi) documentation.

Along with that, the general collaboration of AI involved IoD is critical. In this integration approach, machine learning techniques play a vital role to manages these highlighted prospects of data optimization with a secure protocol for drone-enabled data management and exchange over the network. The list of algorithms that readily associate with the current working procedures of cybersecurity to enhance the sharing of data privacy, protection preservation, and security is as follows:

(i) Gradient descent
(ii) Adaptive learning rate
(iii) Zeroth order optimization
(iv) Meta-learning
(v) Stochastic gradient descent
(vi) Derivative-free optimization
(vii) Conjugate gradient

Furthermore, to address IoT security and privacy challenges for drones, as IoT-enabled drones continue to expand across various industries, ensuring the security and privacy of these interconnected devices has become a critical concern. The existing cybersecurity solutions and standards often need to be revised to address the unique challenges IoT-powered drones pose. Therefore, a smart cybersecurity architecture especially suited to safeguarding and securing IoT-enabled drones is urgently needed. A comprehensive and robust framework that integrates cutting-edge technologies, authentication procedures, encryption protocols, intrusion detection systems, and incident response capabilities customized to the unique needs and vulnerabilities of IoT drones is lacking in the current environment. IoT drones are vulnerable to interception, intrusion, unauthorized access, data breaches, and physical manipulation without an appropriate cybersecurity framework, which can have serious repercussions like compromised privacy, data loss, operational interruption, and even safety issues. By creating and implementing a smart cybersecurity system that includes encryption and secure communication, authentication and access control, firmware and software security, and intrusion detection, this study seeks to overcome these difficulties.

The following sections in this research are arranged as follows. Section “Literature review” presents background works and highlights the previous model. Section “Proposed Framework” discusses the methodology used in this paper. Section “Experiments and results” presents the experimental evaluation and points out the existing gaps in the reviewed literature and insights for future research, while Sect. “Conclusion” presents the conclusions.

## Literature review

Law enforcement agencies use technology far more frequently in their daily operations. The most recent advancements in information technology and digital forensics enable more effective and efficient use of real-time monitoring, drone technologies, criminal tracking, crime investigation, spying, and bugging. In a particular situation, the technology-based solution outperforms human police officers when AI is utilized for crime suspect analysis and detection. The same technologies can be used to monitor and assess the environment at the target location to improve safety and reduce crime. To find potential criminal activities, it uses ML algorithms. AI can be used to warn the public or local law enforcement officials of potentially upsetting circumstances^9–11^. Drones are widely used and applied for military and defense purposes^12, 13^. Drones come in various sizes, from military drones 200 feet to small flying machines. Drone size is an essential factor in terms of utilization and functionalities^14,15^. A military drone with a 16,999-mile range can cover much ground in a short amount of time^16^. The surface area, surroundings, and altitude affect the maximum areal duration^17^. The current research reveals that between 2015 and 2021, 51 security-related publications were included in the Web of Science (WoS) database^18^.

### Drone security threats

There are several categories for drone security presented which is based on their size, usage, and control mechanisms. Undoubtedly, the drone uses the Wi-Fi communication protocol (IEEE 803.11) entirely for the purpose of communication^19^. The drone infrastructure includes a terrestrial hub and Wi-Fi network, subject to cybersecurity threats. According to Yin et al., the equipment's lack of encryption methods makes drones vulnerable to hijacking^20^. According to Koslowski et al., hijackings may result from assaults such as man-in-the-middle with a 3 km broad range^21^. IDT is growing in popularity in the military industry, as demonstrated by Ozmen et al.^22^. The fact that it was made to protect security and privacy is one of the main problems. Khan et al. demonstrated loss, cryptographic techniques, and data protection as significant privacy problems^23^. Several researchers have recently discovered security concerns, including protocol-specific, corrupted components, and sensor-specific threats. Prior research has focused chiefly on detecting drone cybersecurity issues. The prevention of these dangers is frequently overlooked. Ranjitha et al. proposed cryptographic data while transmitting to a terrestrial terminal using a secure encryption method^24^. According to Li and Bai^25^, mini drones have recently attracted much academic interest because of their small size and lightweight. Government and public information privacy are at risk due to the small drone. Numerous other studies, like those by Tuli et al.^26^, Cabassi et al.^7^, and Aldhyani et al.^3^, have examined the risks and problems that drones represent in terms of security. An efficient and clever edge-assisted IDT authentication approach was provided by Khan et al.^27^ to secure the IoD. Drone security monitoring architecture for a manufacturing setting was presented by Maghazei et al.^28^. Kapoutsis et al.^29^ suggested a framework for gas-emission industrial drones. In the security and agriculture sectors, drones are mostly used for monitoring. Examining drone cyber threats has been a problematic research area for the past 10 years. Smart city drone applications and the associated privacy problems were covered by Nguyen et al.^30^. Kumar et al.^31^ and Aydin et al.^32^ addressed drone networks' limits and future directions, and cybersecurity risks. In addition to problems and solutions, Aloqaily et al.^2^ noted security vulnerabilities associated with commercial and industrial drones. IoT-based drones for agriculture were taken into consideration by Saha et al.^33^. According to Lyu et al.^34^, commercial drones must deal with issues such as drone data theft, UAV theft, and drone hijacking. Jares et al.^35^ provided remedies and responses to security-related problems. GPS spoofing is also a problem with UAVs and needs an authentic, efficient, and safe solution. Several attempts at controlling and hacking UAVs were described in detail by Talaei et al.^36^.

### The challenges, issues and vulnerabilities of UAVs

No wireless security or policy standardization is available for these UAVs^37–39^. As seen in Table 1, this results in several dangers. Researchers have also tackled various cyber-attacks related to several types of UAVs in a pre-controlled environment^40–45^. The crash of drones with numerous parallel queries and the alteration of the request packet is called the buffer-overflow attack. However, some researchers used the cache-poisoning strategy, which resulted in the drone and GCR contact being cut off. In every situation, most attacks target the drone's microcontroller or operating system^46^. Technological advancements have made UAVs increasingly susceptible to such attacks^47–53^. GPS spoofing is the most prevalent type of attack, including zero-day attacks, signal jamming, and de-authentication. The Authentication, Authorization, and Accounting (AAA) framework defines the criteria for drone operation in any location. It grants several privileges to the controller of a drone to operate by the administrative rights mentioned while also establishing some stringent authentication procedures for drones to safeguard drone control so that it cannot be transferred to an unidentified third party. Furthermore, the operator can be quickly identified if there is any doubt or illegal action by drone. This reduces illegal spying, privacy issues, and cyberattacks. As a result, numerous mechatronic engineering methods have been proposed to counteract these harmful operations^54^.

**Table 1:** Summary of related work of smart cybersecurity.

Vulnerability type	Description
Spoofing	These are flaws with the communication technique and serial port connections that need to be adequately encrypted^44^. Because of this spoofing issue, GPS information can be captured and manipulated
Malware issue	In several instances, these UAVs are typically connected to and operated by cell phones or any remote control. These methods are not secure^43^; therefore, it is simple to hack UAVs by injecting a reverse-shell TCP payload into their memory. Additionally, this permits malware to be automatically installed over UAVs
Physical design and control system constraints	Designing the control system for unmanned aerial vehicles (UAVs) encounters various challenges. One significant obstacle is the slow convergence rate, which hinders the drone from executing rapid or forceful maneuvers. As a result, flaws in-flight performance and deviations from the intended trajectory may become apparent^47,59,60^. This sluggish convergence rate and occurrence of glitches can be attributed to either the physical design of the drones or the underlying control system, which primarily aims to maintain stability in unpredictable environments
Manipulation and other common concerns	The flying pathways that UAVs must follow are pre-programmed, so they can be changed ^45^. However, the most frequent problems are caused by wind, overheating, or any predator bird that could easily destroy the small, lightweight drone^46^
Wi-Fi Constraints	It can be dangerous to use Wi-Fi to operate drones. This is demonstrated in Ref.^48^, where the links were harmed using software and altered UAV control
Sensitization issue	It has also been demonstrated that ultrasonic waves may attack the MEMS gyro sensors on these UAVs because they are sensor-dependent^47^
Firmware issue	The prototype's and algorithm's flaws are exposed after use^50^
GPS issue	The GPS module, which is sometimes not secured and might result in spoofing, is what the automatic reliant surveillance broadcast relies on Ref.^49^
Controller issues	These problems with the operation control unit may perplex the controller by switching the live feed to a different video^52^
Sky Jack-based attacks	One piece of software used in attacks involving the de-authentication of targets while in control is Skyjack^51^

These drones are inexpensive and widely accessible in marketplaces; thus, criminal conduct can be carried out using them. They are more dangerous because of their ability to carry a range of external payloads, which could lead to drones carrying explosives or toxic chemicals. Furthermore, their capacity to reach locations where normal humans cannot make them more harmful because they can deliver anything without drawing attention^55^. It should be mentioned that safety is a concern as well, especially if drones are flying in overpopulated areas and crash owing to a variety of defects^56^. These kinds of instances have frequently been reported. One of the instances occurred in April 2016, when a UAV collided with a British Airways BA727 passenger plane. After reviewing these incidences and issues, the following public safety measures can be implemented: A drone will likely be hacked or diverted from its intended direction due to strong winds. Therefore, a reset button should be accessible to return the drone to a hovering state and aid in regaining control. Some places where drones may encounter signal jammers and then be managed for a cyberattack. As a result, drones must include some form of sensor that can identify signal jammers in the area.

### Drone security using machine learning

The three most common ML technique types are semi-supervised, supervised, and unsupervised learning. To combat cyber threats in IoT networks^57^, cloud computing^58^, and communication networks^59^, several researchers have employed ML models. To identify DDoS assaults using two characteristics, A self-adaptive model using RF and LSTM was integrated with a learning strategy by Vedula et al.^60^ (autoencoder). Hosseinzadeh et al.^61^ developed a probabilistic approach in a restricted cyber-physical system for identifying and managing an actuation danger. Only a little research has been done on ML-based assaults on drone networks. The current research prominently suggests an access control technique for drone security.

Table 1 summarizes the most current studies on using ML in wireless security network solutions. A thorough literature review revealed many publications addressing privacy and safety issues with drone data security between 2010 and 2020. Most of the study examines cybersecurity challenges, uses, and problems. Additionally, spoofing, drone hijacking, and data protection are considered. Several research studies have recognized the problem domain, but workable solutions have yet to be provided. Bera et al. put up a solution based on blockchain for data security^62^ during communication through IoT-enabled drones. Manual attack detection is a component of the described approach. Drones based on IoT networks were proposed. However, a device-based authentication mechanism was not appropriate for it. The development of a safe IDT presents an open research issue by proposing a method that solves concerns about cybersecurity threats to guarantee drone flexibility in the manufacturing industry.

A complex and smart framework is required for drone security to analyze data from assaults and guarantee drone security by implementing appropriate activities. In the past, mobile-based networks for the defense against cyber-attacks have been suggested using artificial intelligence-inspired methodologies, but drone-based security has yet to be included. The approach for drone authentication, security, and access management proposed in the current study is motivated by machine learning.

### Drone security using deep learning

Neural networks are used in deep learning, a modern field of AI. These neural networks are more accurate classifiers and predictors because they have more hidden layers^63,64^. DL algorithms have numerous applications in present smart cities due to their ability to tackle problems with incredible skills and efficiency. The authors thoroughly examined the application of DL in upcoming smart cities^65,66^. The topics explored in this study are smart mobility, smart city urban modeling, transportation, intelligent infrastructure for smart cities, smart urban governance, smart education, smart health solutions, resilience and sustainability, and smart urban governance. Concerns about privacy and cyberattacks have increased because of the growth of smart devices and interconnectivity through IoT. The DL algorithms are highly effective in dealing with cyber threats due to their exceptional anomaly identification and categorization skill. The researchers used several DL algorithm-based techniques^67–74^ to identify and counteract cyberattacks on the IoT-based infrastructure used in smart cities. These results also point to potential topics for future research by comparing the accuracy of various DL algorithms. Deep hierarchical models and deep learning models have proposed the learning of non-linear correlations between data for malicious attack detection^75,76^.

The VIRAT2020 dataset is utilized to detect intrusions using ANN, which lowers features from correlation and data gain^77^. The accuracy of the results from the model was increased. The author combined multivariate component analysis and PCA, offering a method for detecting DDoS attacks in a real-time approach^78^. Based on the trustworthy and current CICIDS2017 network attacks dataset, Musafer et al.^79^ developed a sparse classifier for systems that detect intrusions. The NSLKDD and KDD CUP 99 datasets were used to evaluate the authors' suggested deep learning model, which uses a memetic algorithm to identify unusual traffic^80^. To create an effective system for detecting intrusions, feature augmentation has been combined with SVM and has produced reliable results regarding false alarm rate and training speed^81^. Researchers have used multilevel intrusion detection to detect intrusions^82^. A unique neural network model has been put out for intrusion detection to increase accuracy^83^. Additional hazards and security difficulties are brought about by expanding network connectivity and integrating terrestrial networks with satellite networks. DDoS is one of the most frequent attacks that disrupt service in satellite-terrestrial integrated networks. Numerous research has been put forth in the literature to identify DDoS in satellite and terrestrial networks.

## Proposed framework

The proposed UAV Framework utilizes a hybrid ML and DL approaches for Intrusion Detection (IoD) in UAV networks. It is designed to accommodate the structure of conventional networks where drones connect with base (drone) and ground base stations for transaction management. The framework consists of two main components: the base and ground station, both responsible for capturing and processing data. Unlike traditional networks that can rely on a centralized module, the proposed framework for drones may require separate hybrid modules for the base station and ground station. The base station module controls all drone communications and validates the selection of the drone's module. Distributed modules are employed to detect and assess the level and type of attacks. Each drone is equipped with a module that directly monitors attacks on the drone, while a second module is situated at the ground base station. These modules collaborate to validate attacks and determine which drones should be notified. All drones in the sky can communicate with the base station, a single station, or a network of stations. Streaming or batching for drone intrusion detection depends on the technology used. Batch processing is required when employing MapReduce as a significant component for decision-making, as it requires time for development. However, runtime identification can be performed using frameworks like Flink, Storm, Apache Kafka, or Spark. In this study, Apache Kafka is preferred due to its efficient handling of massive data streams, particularly during the initial stage. The study simulates real-time analysis by providing data as a stream to the modules. Figure 1 illustrates the Smart Framework Layered Architecture of Drone Attacks. The two primary components of the framework are drones and base stations.

### Hierarchy of the proposed model

#### Drone layer

The drone layer comprises a camera-equipped quadcopter, the initial layer in the proposed tiered architecture for industrial drones. IoT sensor data update this layer. A camera, GPS sensor, radar, and altitude sensor are deployed as smart sensors. In the suggested architecture, this is the initial stage. This layer can sense, record, and communicate the data collected via drones to the layer above. An unmanned aircraft system (UAS) drone is applied at this layer, which oversees drone flight operations, sensor data logging, etc. The ground controller and the communication connection comprise the two components of the UAS. The disclosed design uses a DJI Phantom 3 drone with a special communication link. and remote control. The drone is equipped with sensors according to the suggested architecture.

#### Edge processing layer

**Figure 3:**
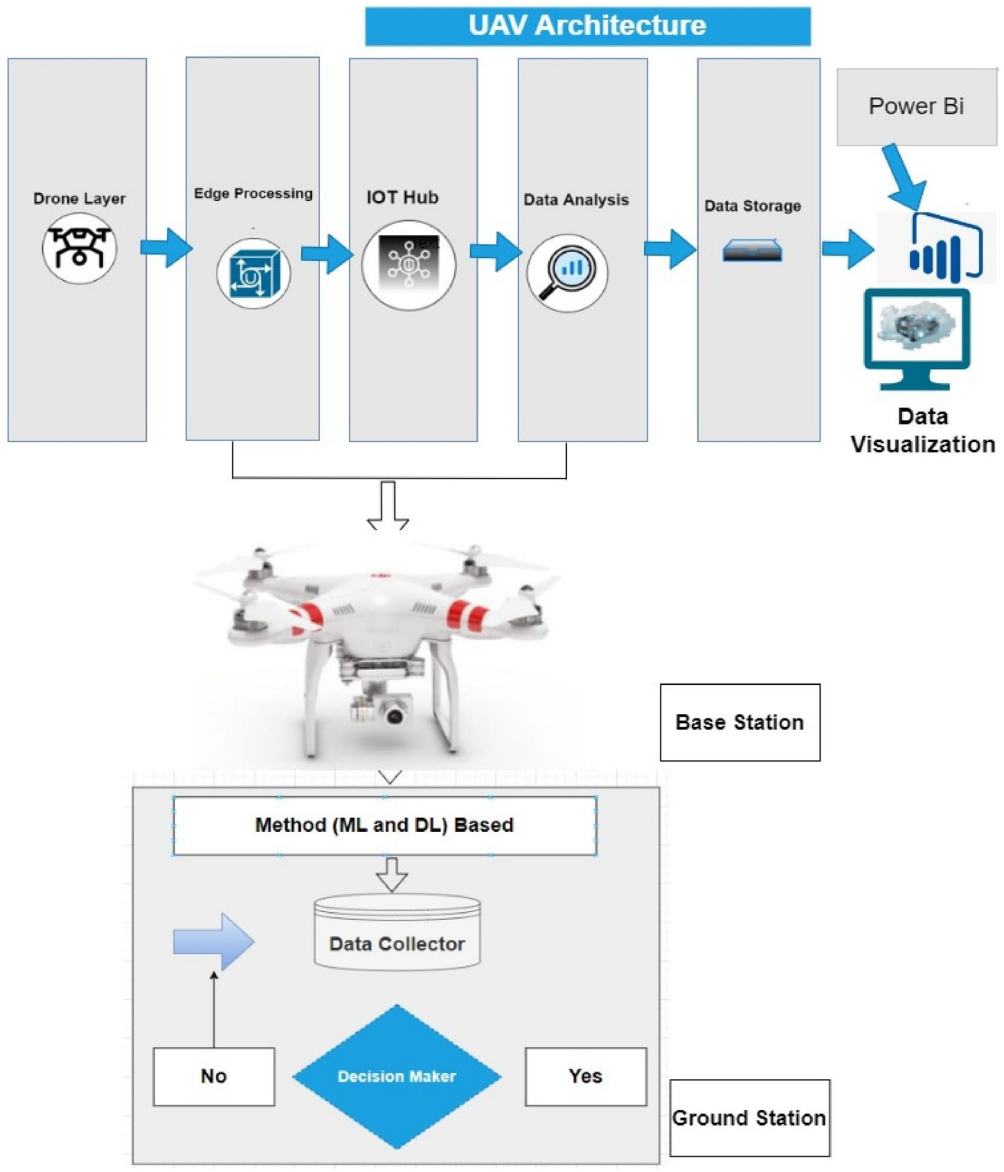
Smart Framework Layered Architecture of Drone Attacks.

The privacy and security layer at the second layer receives the data from IoT and drones, known as the edge processing layer for the Internet of Drones (IoD), where the data source is verified as being from approved sources. This layer corresponds to the cloud layer and is responsible for data transmission and communication. Numerous gateway device methods enable wireless communication. Information is transmitted quickly using Wi-Fi connectivity. The edge processing layer efficiently facilitates communication between devices and the cloud. This layer controls flooding, cashing, and data protection. The Azure IoT gateway is implemented for cloud connectivity in the proposed research. Figure 3 depicts the design of the IoT gateway.

#### Security and privacy layer

The following layer utilizes machine learning models to provide device authentication and safe access control. The main component of this IoT framework, data safety, and security, is implemented at this level. At this point, numerous threats to privacy could emerge. They are 1. Physical threat to privacy; 2. behavioral threat to privacy; and 3. location threat to privacy. Taking possession of someone's property is connected to physical privacy. The privacy of someone's possessions may be threatened if someone else is covertly keeping an eye on the drone data. An individual's location being recorded by an unauthorized person is a location privacy threat. An unauthorized party watching someone's actions and conduct is considered a threat to their privacy. Authentication procedures and schemes must be used to combat these kinds of security concerns. Unauthorized individuals make such security threats through a variety of security vulnerabilities. The most prevalent threat types include spoofing, DoS, jamming, and intrusion attacks. An algorithm that uses machine learning to detect and alert users of this kind of vulnerability is used to ensure device authentication in the proposed architecture.

#### Device connection layer

**Figure 4:**
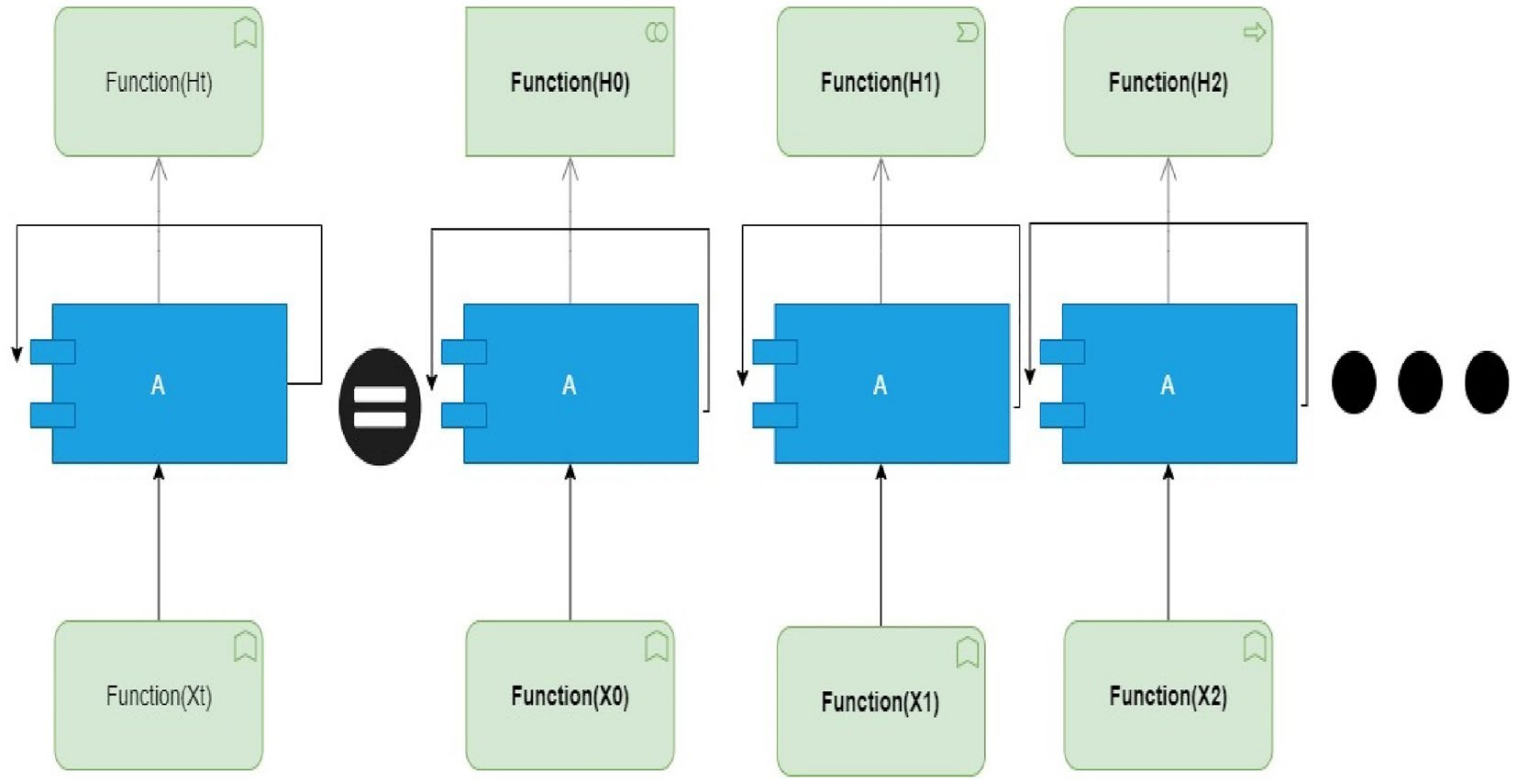
Working of Hybrid ML-DL.

IoT gateways are essential for connecting to a base station's cloud-based IoT Hub. A further module for security orchestration and automation is included in this case to guarantee connectivity for only authenticated devices. The IoT Hub acts as a messaging intermediary between IoT devices and applications. The IoT hub in an IoT network enables communication between IoT devices and cloud-based platforms. It is a two-way conversation. Only authenticated devices are subject to the security mechanisms at this layer. The procedure for registering and encrypting network-connected devices is shown in Fig. 4. The blockchain client receives sensor, drone, and network data, protects the data's integrity, and saves the data in a database on a cloud server. Real-time security for IoT devices is provided through primitive blockchain technology.

#### Data processing layer

This layer receives the data from the IoT Hub and uses it to evaluate the drone's data stream. In this case, two new modules are put into use: a data hub service that facilitates easy and convenient cloud storage and a machine intelligence component that analyses data intelligently. Following the circumstances and needs of the data, a variety of machine learning algorithms are available. This study aims to develop an intelligent machine-learning strategy for device authentication. This layer comprises an authentication system built using the clever machine learning method Naive Bayes. The IoT hub layer uses drone timestamp data for a set period to authenticate devices. The model is developed and validated using data from drone flights. The model is first trained, then testing is done to see if the model is smart enough to recognize malicious drone activity. The model will notify the system and prevent the device from connecting to the cloud if the drone information is erroneous. When a drone behaves inappropriately, it is promptly identified, and machine intelligence is used to prevent unwanted access. Several security risks accompany flight operations. The most frequent threat is a man-in-the-middle assault, which happens when a third party takes control of the drone. False information may also spread when an unauthorized individual attempts to run the drone. The Naive Bayes classifier is implemented in the proposed architecture to train a model, which is subsequently used to validate freshly generated aircraft paths. We calculated the precision, recall, and accuracy using the real-time and VIRAT2020 datasets. Recall is the percentage of inaccurate forecasts, while precision is the percentage of accurate and accurate predictions.

#### Data storage layer & data visualization layer

**Figure 5:**
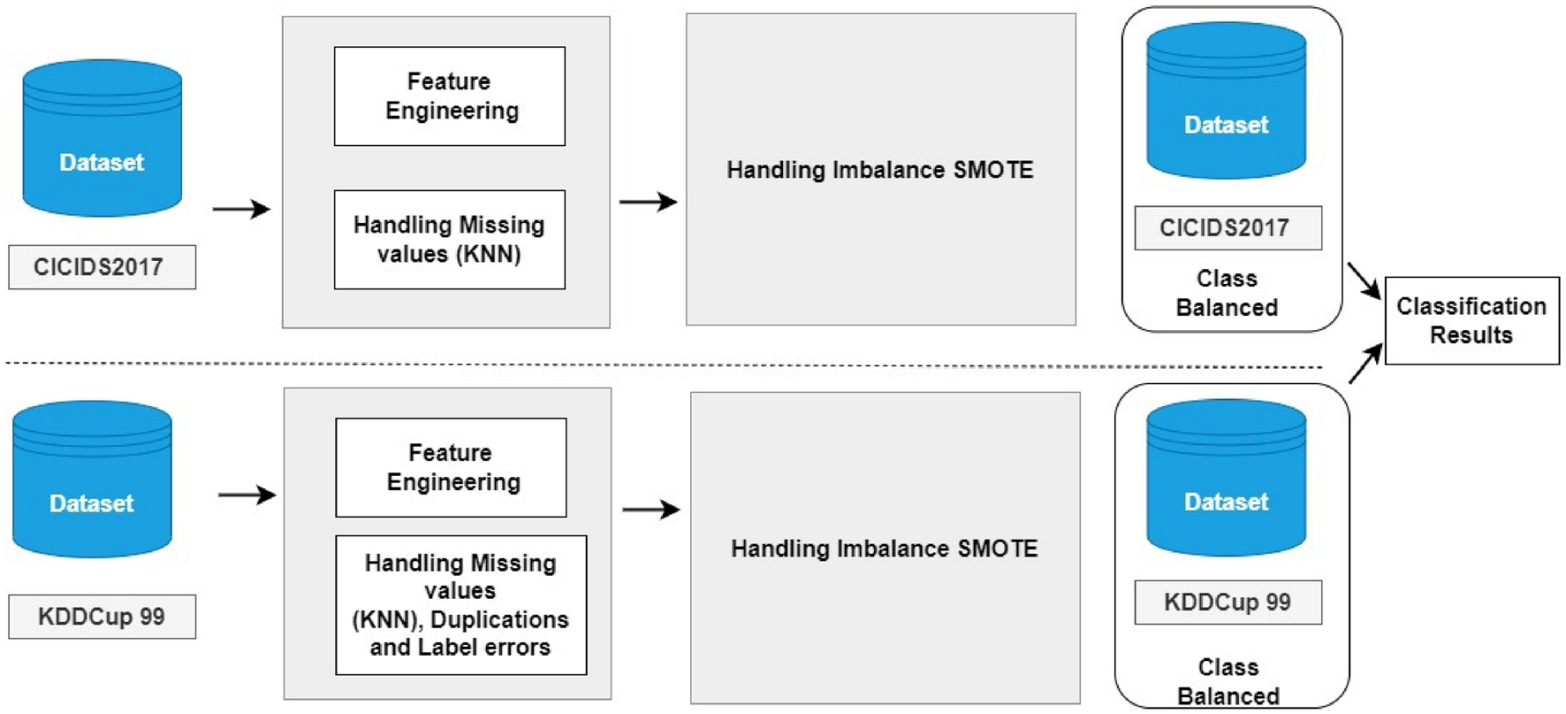
Bias variance.

**Figure 6:**
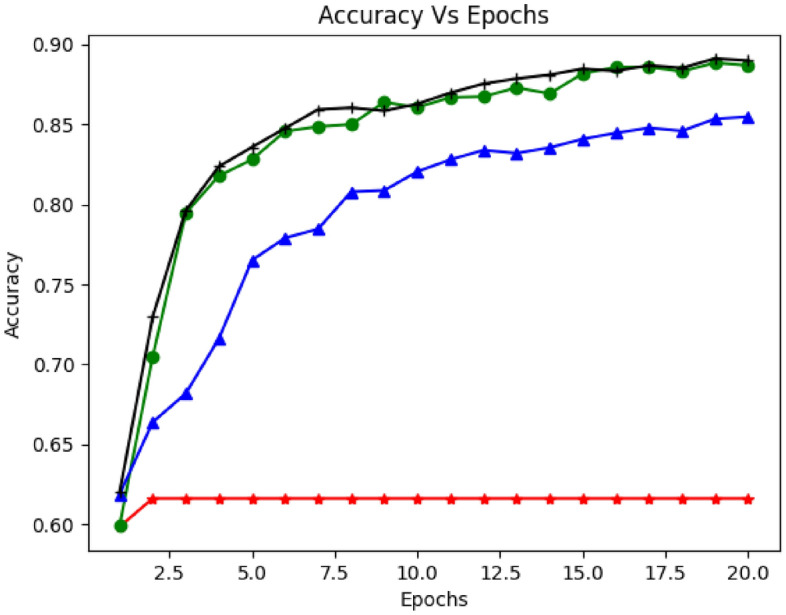
Accuracy vs. Epochs based on Recurrent Neural Networks (RNN).

The data storage centers at the data storage layer are where the outcomes of the data processing produced by the data processing layer are kept. The drone layer stores the results drones produce in a cloud-based NoSQL database. The information consists of IoT sensors, a network, and drones. Data may be easily accessed and retrieved due to the schema-less storage offered by NoSQL databases. This method allows for the storage of many data. As a self-referential database, a NoSQL database is more practical than a SQL database. These databases often use the storage structures depicted in Fig. 5. The most popular structures are displayed, including documents, graphs, key-value, and columns. The layer of data visualization enables a variety of tools and services for data monitoring. This platform uses Microsoft Azure services for hub services and storage services. The findings produced by the visualization layer, which displays the forecasts made by our intelligent model about the security level of a drone, are seen through a mobile app. The Nave Bayes algorithm is used to detect drone attacks. Using the findings of stream analytics, which are kept in a storage center, Fig. 6 illustrates the architecture of business intelligence. Power BI, a business intelligence modeling and result visualization platform uses these findings.

### Hybrid drone security

IDSs must have a deep understanding of all past attacks that have been found. Statistical methods only work effectively in a drone system open to unexpected threats. Unsupervised learning algorithms are enhanced strategies to detect attacks based on device data and generate alerts about unusual attacks. The gadget could spot irregularities and take precautions against attacks in this approach. When the defense system fails to stop an assault, the gadget raises alarms, alerting the system administrator. This provides the primary distinction between learning-based intrusion detection systems and signature-based systems. However, most attacks will only be noticed if there is previous knowledge. Additionally, data noise may affect the detection process. The effectiveness of the supervised and unsupervised tools has improved due to advancements in deep neural networks.

#### IoD with ML

sIn the domain of drone Intrusion Detection (IoD) within UAV networks, various Machine Learning (ML) methods have been explored to detect and mitigate potential attacks. This section discusses some commonly employed ML methods, including Logistic Regression (LR), Decision Trees (DT), Random Forests (RF), and Naive Bayes, for drone IoD.

##### Logistic regression (LR)

LR is a widely used ML algorithm for binary classification tasks. In the context of drone IoD, LR models can be trained on labeled datasets to classify network traffic as either normal or malicious. LR excels at providing interpretable results by estimating the probability of an instance belonging to a specific class based on feature weights. It can serve as a baseline method for initial drone IoD experiments.

##### Decision trees (DT)

By building a hierarchical structure of decision rules based on the input features, DT algorithms are tree-based machine learning techniques. DTs are simple and can capture complicated decision boundaries. In drone IoD, DTs can be trained to identify malicious or benign network traffic based on criteria such as packet headers, payload properties, or communication patterns. They are adaptable for identifying different kinds of drone assaults since they can handle both continuous and categorical data.

##### Random forests (RF)

Various decision trees are combined in the RF ensemble learning technique to increase prediction resilience and accuracy. RF models are particularly good at handling noisy data and high-dimensional datasets. RF can employ an ensemble of decision trees trained on various subsets of the data to categorize network traffic in the context of drone IoD. This ensemble approach improves the intrusion detection system's overall performance and robustness.

##### Naive Bayes

The probabilistic ML algorithm Naive Bayes is based on the Bayes theorem. It determines the likelihood that an instance belongs to a particular class under the assumption of independence between features. Large datasets can be handled by naive Bayes classifiers, which are also computationally efficient. In drone IoD, Naive Bayes models can be trained with labeled data to determine whether observed feature patterns in network traffic indicate benign or malicious activity. Despite the erroneous feature independence assumption, naive Bayes can produce surprisingly good results in practice.

#### IoD with DL

Machine Learning (ML) techniques that use recurrent neural networks (RNNs) in the field of drone intrusion detection (IoD) within UAV networks have shown promise in identifying and thwarting possible attacks. The RNN versions of Gated Recurrent Units (GRU), Recurrent Neural Networks (RNN), Long Short-Term Memory (LSTM), and Bidirectional LSTM (biLSTM) that are frequently used for drone IoD are covered in this section.

##### Gated recurrent units (GRU)

A form of RNN design known as GRU solves a few drawbacks of conventional RNNs. GRU models are better at capturing long-term dependencies in sequential data because they feature gating mechanisms that enable them to update and reset their internal states selectively. In drone IoD, GRU models can examine network traffic patterns over time while considering the previous context to categorize occurrences as legitimate or malicious. They are useful for real-time assault detection in UAV networks because they are computationally efficient and can manage temporal dynamics well.

##### Recurrent neural networks (RNN)

RNNs are a subset of ML models created especially for processing sequential input by preserving hidden states that store knowledge from earlier time steps. RNNs are suitable for drone IoD because they can detect temporal dependencies in time-series data. To analyze the temporal patterns in network traffic and spot anomalies or malicious activity, RNNs can be trained using labeled datasets. Standard RNNs, however, could experience the vanishing gradient problem, which hinders their capacity to detect long-term dependencies. The nodes in Recurrent Neural Networks (RNN) connected are one of the deep learning techniques. These nodes can handle input and output individually, even though each data element is handled separately and stored in sequential order. RNNs are useful in various tasks, including video processing, time series prediction, natural language processing, and speech synthesis. Figure 2 illustrates the multi-layer perceptron design used by RNNs. Additionally, it has a looping design that acts as the primary pathway for information transfer from one level to the next. The extracted RNN loops are displayed in Fig. 3 as folded RNN layers.

##### Long short-term memory (LSTM)

LSTM is an RNN variation incorporating memory cells and gating techniques to solve the vanishing gradient issue. LSTMs can effectively capture long-term dependencies in sequential data by selectively storing or forgetting information. In drone IoD, LSTM models can recognize hostile behavior and understand intricate temporal patterns in network traffic. They are very helpful when long-range dependencies are crucial for spotting complex attacks.

##### Bidirectional LSTM (biLSTM)

A variation of LSTM that processes the input sequence forward and backward, biLSTM incorporates data from previous and upcoming time steps. Thanks to this bidirectional processing, the model may capture a more thorough grasp of the context and dependencies in the data.

It is crucial to remember that the effectiveness of these ML techniques, such as GRU, RNN, LSTM, and biLSTM, depends on several variables, including the accessibility and caliber of labeled training data, the complexity and variety of attack patterns, and the unique features of the UAV network. After careful analysis and trial, the best ML strategy for drone IoD in each situation must be determined. Additionally, combining these techniques with other ML algorithms or ensemble techniques can improve the precision and efficacy of drone intrusion detection systems in UAV networks.

#### Drone data collector

The data collectors gather the RNN-LSTM module information. This module is also in charge of splitting the data packets into their parts and extracting parameters like reception rate, source IP, transmission-to-reception ratio, transmission rate, destination IP, duration of the activity, and transmission mode. The data collector is given this responsibility since, as was already indicated, our architecture is built to work for batch and stream data modes. As a result, two collector modules are suggested in our architecture, one in each drone component and the other in the base station component, as shown in Fig. 1. The collector configured that buffer data when analyzing batch data. The data collector will oversee providing the data to the RNN-LSTM module in stream form when using the data stream mode. It was the method used in this investigation. The data collector simulates real-time data processing and adjusts the data as necessary because we are replicating the drone's activities.

In contrast, the data collector in physical drones, which is not the case in this work, will oversee intercepting the data from the communication module and preparing it to meet the needs of the RNN-LSTM module. The module is furthermore in charge of sending the RNN-LSTM module's decision and the data it has gathered to the base station collector module. All the drones' data and decisions are sent to the base station data collector module. It analyses all incoming data for decision verification and sends it to the base station's central RNN-LSTM module. The decision-maker module will then get the conclusion and proceed with further processing. The hyperparameters of the proposed framework are shown in Table 3 with (Units, batch size, epochs, dropout, batch size, and optimization). We use a minimal dropout value of 15–35% of neurons during training, with 20% serving as a decent starting point and teaching neurons how to identify attacks. A probability that is too low has little impact, and a probability that is too high prevents the network from learning enough. Moreover, epochs deploy drone assaults following the performance. Even while training accuracy improves, increase the number of epochs until the validation accuracy declines (overfitting).

#### Mitigating bias and variance in data

In this section, we addressed the limitations presented in Fig. 5 of KDDcup 99 and CICIDS2017 datasets. The KDD Cup 99 dataset's substantial redundancy, which might induce bias throughout the learning algorithms, is a serious negative. This bias tends to favor frequent records while impeding the learning of uncommon ones, which are often more destructive to different network attacks. In addition, the inclusion of these repeating records in the test set may influence evaluation results in favor of techniques that have higher detection rates for common data. To resolve this problem, we carried out a comprehensive data cleaning procedure, removing all duplicate entries from the KDDCup 99 test, and training sets and keeping just one copy of each record. The decrease in duplicate data for the KDDCup 99 test and training sets. We identified several constraints while analyzing the features of this CICIDS2017 dataset. One glaring drawback is its size, spanning eight files and encompassing five consecutive days of traffic information collected by the Canadian Institute of Cybersecurity. Building a realistic Intrusion Detection System (IDS) would be more feasible with a single, consolidated dataset. Additionally, the dataset contains a significant number of redundant entries that may not be crucial for training an IDS. We also observed a severe class imbalance problem within the dataset, despite its relevance to contemporary attack scenarios. Such class imbalance can mislead the classifier and bias it towards the dominant class. To address the issue of scattered data across multiple files in CICIDS2017, we consolidated the data. Furthermore, missing values were removed. While the dataset's substantial volume presents a limitation, it is inherent to typical datasets containing comprehensive information. The challenge of high volume can be mitigated by sampling the dataset before initiating the actual detection process. However, it is crucial to emphasize that addressing the class imbalance issue is a prerequisite. Balancing the dataset increases the likelihood of instances from all class labels occurring, enhancing the overall effectiveness of the analysis. IDS within wireless sensor networks can be framed as a classification problem, involving the categorization of data into two categories: normal data and attack data. Addressing the issue of class imbalance between these two categories, and seeking to enhance classification accuracy, involves the utilization of SMOTE (Synthetic Minority Over-sampling Technique). SMOTE is employed to increase the representation of the minority class by generating synthetic instances, effectively rebalancing the dataset. Consequently, this rebalanced training set improves the model's ability to tackle the inherent class imbalance within the original data.

#### Sensors and transmissions

Table 2 Hyperparameters proposed framework with RNN, LSTM, and Bi LSTM.

The ZigBee wireless technology is used due to the characteristics, analogies, and capability of digital information transmission. The proposed framework utilized XBee Pro S1, which can send data over a great distance. The data is collected with the following sensors.

- Sensor GPS
- Radar Sensor
- BMP180 Pressure Sensor

##### Device GPS

The NEO-7N chip and an electrical circuit make up the GPS receiver known as the GY-GPS6MV2. An LED display and a battery make up its construction. The light comes on when it sends GPS data across satellites. This sensor module also has an approximate 161 dBm sensitivity. Radar Detector: This is used to monitor and recognize items far away. These sensors emit electromagnetic radiation in the direction of targets and objects. Compared to optical sensors, these sensors offer enhanced accuracy in identifying objects. Radar sensors can be replaced with accelerometers in the proposed system. Specifically, an HC-SR04 ultrasonic proximity sensor is utilized. Radar sensors are employed to calculate object patterns. The BMP180 Pressure Sensor is employed for altitude and pressure measurements, which consumes minimal battery power. It is compact and exhibits excellent precision. The pressure sensor module is factory-calibrated, ensuring superior accuracy compared to other sensor alternatives.

#### Drone data centralized RNN

On the base station, in this instance, another RNN-LSTM is deployed. Again, this module might operate on streams or batches. According to the selected mode, it receives drone traffic from the data collecting module either in streams or in batches. To determine which drone is compromised, the central RNN-LSTM will decide based on the total amount of data gathered. The decision-maker module receives the decision from the central RNN-LSTM module. Due to the traffic generated by the many drones, the centralized RNN has more training than the RNN on individual drones.

## Experiments and results

In this section, we used impartial measurements to assess the effectiveness of the suggested framework. For statistical parameters, accuracy, precision, recall, and F-measure, we computed temporal efficacy, statistical performance, reliability, and stability results. The outcome for a mobile system is shown, and it includes the drones' security status and an IoT-enabled network with ML and DL. Four assessment metrics were used in the proposed ML framework to assess the model's performance compared to more conventional methods as given in Table 2.

**Table 2:** Simulation Parameters for Proposed ML Framework.

Parameters	RNN	LSTM	Bi LSTM
Units	64	64	64
Batch size	24	24	24
Epochs	20	20	20
Dropout	0.2	0.2	0.2
Batch size	864	1152	1152
Optimization	Adam	Adam	Adam
Training time	2-h 15 min	1 h 48 min	1 h 58 min

The efficiency of these ML approaches for drone IoD may vary based on the network's unique properties, the types of assaults, and the standard and accessibility of labeled training data. This is important to keep in mind. To find the best way to identify and thwart drone assaults in UAV networks, it is crucial to assess and compare various ML techniques carefully. Additionally, combining different ML approaches or using more complex methods like deep learning might improve the precision and robustness of drone IoD systems even more as can be seen in the mathematical equations below.

1 $$ACC\, \left(Attack\right)=\frac{T{P}_{attack}+T{N}_{attack}}{T{P}_{attack}+F{N}_{attack}+T{N}_{attack}+F{P}_{attack}}$$

2 $$PR\, \left(Attack\right)=\frac{T{P}_{attack}}{T{P}_{attack}+F{P}_{attack}}$$

3 $$RE\, \left(Attack\right)=\frac{T{P}_{attack}}{T{P}_{attack}+F{N}_{attack}}$$

4 $$F1 \,Score\,\left(Attack\right)=\frac{PR \left(Attack\right) X RE \left(Attack\right)}{PR \left(Attack\right)+ RE \left(Attack\right)}$$

**Figure 7:**
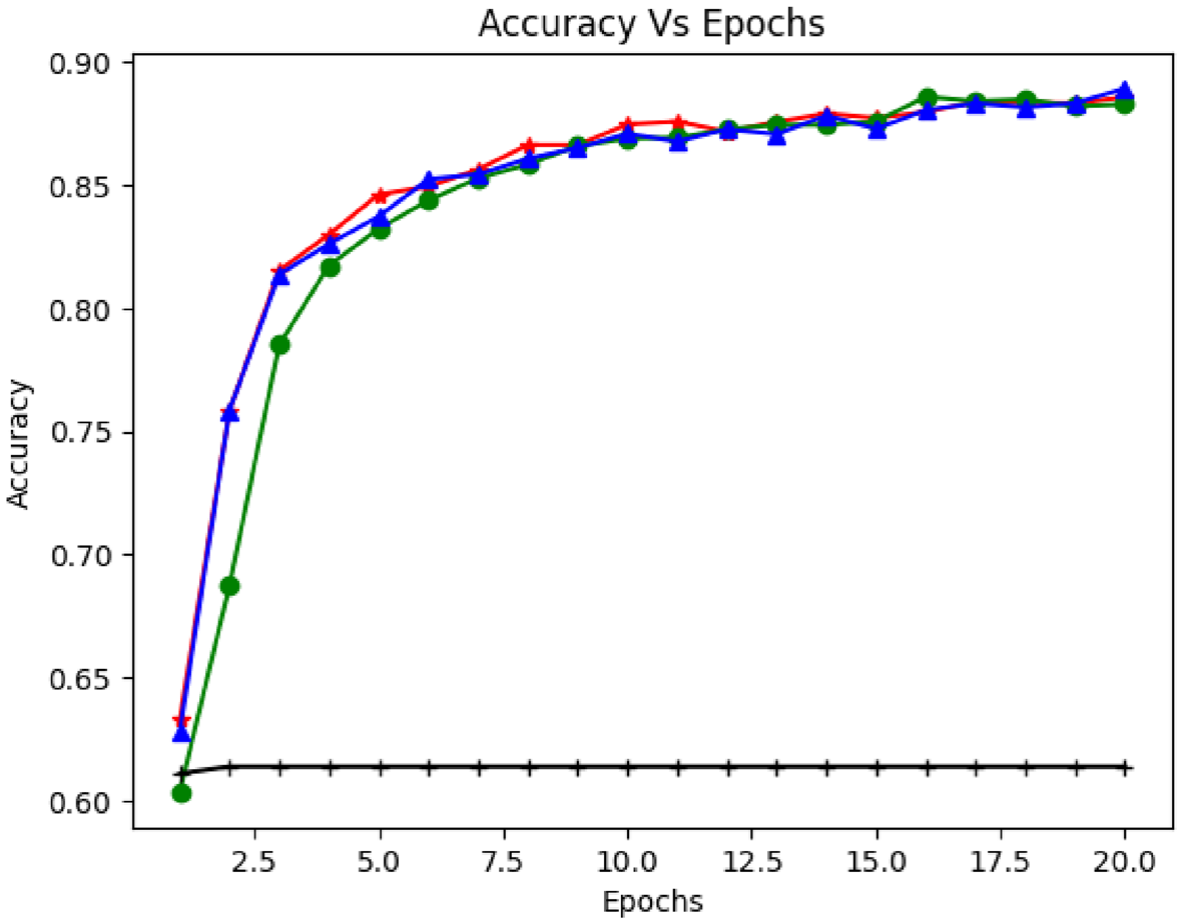
Accuracy vs. Epochs based on Long Short-Term Model (LSTM).

**Figure 8:**
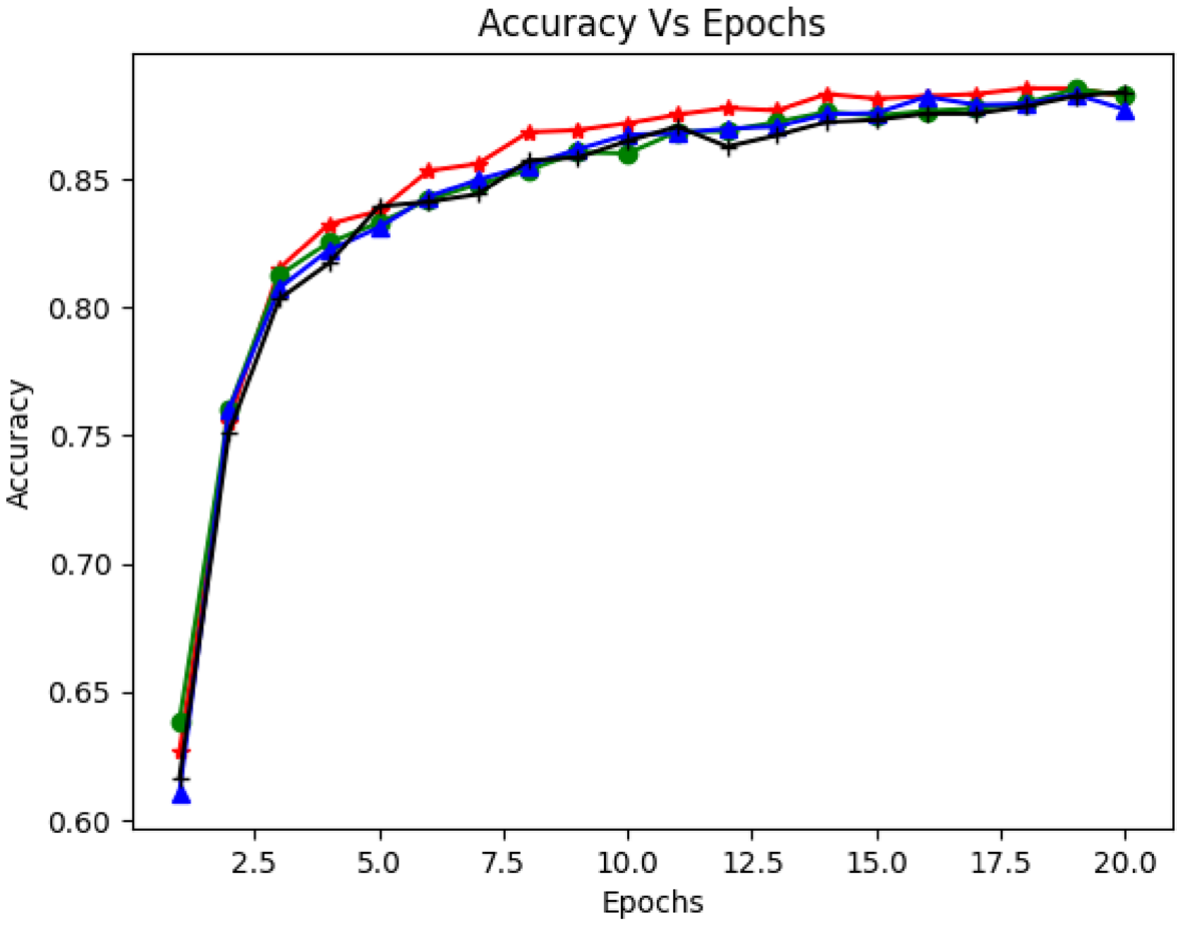
Accuracy vs. Epochs based on Gated Recurrent Unit (GRU).

Figures 6, 7 and 8 demonstrate the model's accuracy with RNN, LSTM, and Bi-LSTM concerning the number of iterations (epochs). The experiment inspected the accuracy of the proposed model with different sample sizes, epochs, and activation functions (Adam, degrade, madam, and Adamax). They push up and down the learning rate of the model. Figure 6 shows the detection accuracy versus epochs. As shown in the graph, LSTM accuracy increased with several iterations. It would be more stable with increased epochs and sample size. Moreover, the average accuracy was (91%) and reached (92%) in some cases. Figure 7 illustrates the model accuracy using a dropout rate of 0.2 along with various activation functions (Adam, degrade, madam, and Adamax). The graph demonstrates a commendable alignment between the accuracy and the actual function. Moving on to Fig. 8, it portrays the accuracy of detection over different epochs. However, when applying the proposed model with GRU and utilizing the relu activation function, the achieved accuracy appears to be relatively lower.

The distribution of normal and attack records throughout 10% validation, 20% test, and 70% training records is shown in Table 3 of this work, along with an overview of the various attacks. The datasets used in this work came from the KDDCup 99 and CSE-CIC-IDS 2018 on AWS, which offer important details on the setups and traits of intrusions. Beginning in 2018, the Canadian Institute for Cybersecurity (CIC) and the Communications Security Establishment (CSE) worked together to create these datasets. To test, analyze, and assess network-based anomaly detection intrusion detection systems (IDS), they set out to create datasets methodically. These datasets provide thorough descriptions of incursions and abstract distribution models for programmers, protocols, or low-level network entities by utilizing the idea of profiles. The datasets capture representations of actual network events and behaviors and provide extensive benchmark resources for IDS. Individual operators can provide network events for various network protocols and topologies because of the profiles' abstract character. The dataset used in this study offers comprehensive descriptions of intrusions aimed against protocols, apps, or other lower-level network elements. It is frequently used to evaluate and test intrusion detection methods. Six different attack scenarios—Botnet assaults, HTTP denial of service, web application attack collection, network infiltration attacks, brute force attacks, and DDoS attacks—are represented in the dataset. Further, details about these attack scenarios can be found in Ref.^29^. 6,437,330 normal records and 1,656,840 attack records comprise the dataset, split into 10% validation, 20% test, and 70% training records. Table 4 shows a detailed breakdown of the various attack distribution types found in the KDDCup 99 and CSE-CIC-IDS2018 datasets.

**Table 3:** Type of attacks.

Dataset	Type of attack	Total number records	(70%) Training	(20%) Testing	(10%) Validation
CSE-CIC- IDS2018	Hulk Attack	461,912	200,333	57,238	28,619
	Slow HTTP Test	139,890	97,923	27,978	13,989
	Botnet-Bengin	762,384	533,668	152,476	76,238
	Slowloris	10,990	7693	2198	1099
	HOIC	686,012	60,208	17,202	8601
	LOIC-UDP	1730	1211	346	173
	Infiltration	161,934	113,353	32,386	16,193
	Brute Force-XSS	1230	161	46	23
	FTP-BruteForce	667,626	135,352	38,672	19,336
	DDos-Benign	360,833	252,583.1	72,166.6	36,083.3
	Bot	286,191	838,860	209,715	838,860
	GoldenEye	41,508	200,333.7	57,238.2	28,619.1
	Web-Bengin	2,096,222	838,860	209,715	838,860
KDD	Benin	60,591	42,413.7	12,118.2	6059.1
	Neptune	58,001	40,600.7	11,600.2	5800.1
	Smurf	164,091	114,863.7	32,818.2	16,409.1

**Table 4:** Comparative analysis of the proposed model with other state-of-the-art.

Methods	Accuracy	Precision	Recall	F1 Score
Random forest (RF)	88.06	85.00	84.59	87.31
Support vector machine (SVM)	86.13	82.02	82.15	82.14
Decision tree (DT)	85.06	82.01	81.06	83.61
Logistic regression (LR)	87.02	83.12	85.20	84.32
Naive Bayes (NB)	80.36	79.01	75.06	70.02
Multiple regression analysis (MPA)	83.30	84.10	84.00	81.20
K-nearest neighbor (KNN)	84.66	80.36	79.36	77.36
Perceptron network (PN)	85.02	**91.36**	**91.36**	**91.36**
Bi-LSTM (Bidirectional long short-term memory)	**91.36**	90.16	90.11	90.19
Recurrent neural network (RNN)	90.16	88.00	89.10	86.00
Long short-term memory (LSTM)	91.00	89.10	89.13	88.11
Gated recurrent unit (GRU)	89.13	82.06	81.00	85.00

Significant values are in bold.

**Figure 9:**
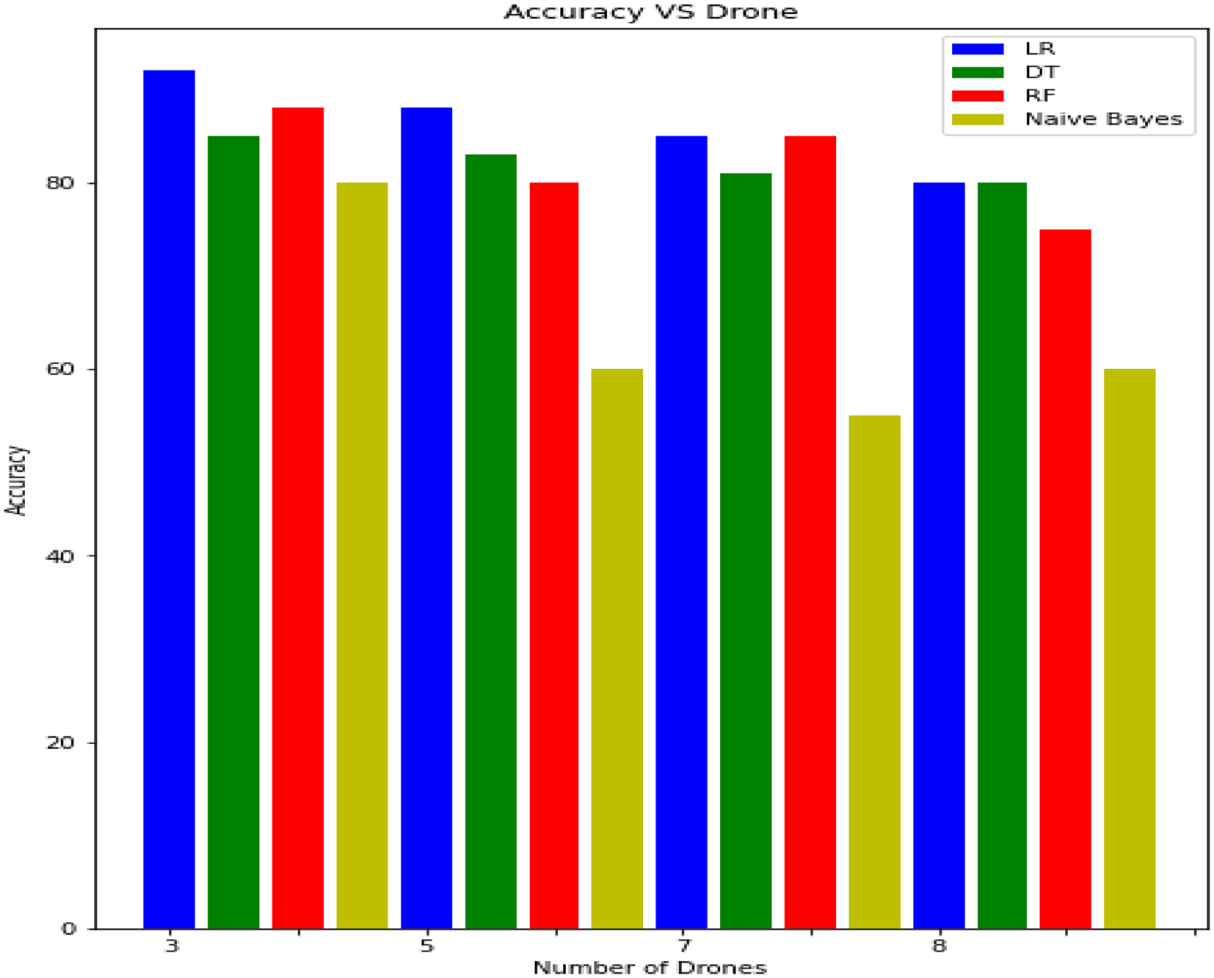
Accuracy Analysis Vs Number of Drones on ML Methods.

Table 4 summarizes the performance of the proposed model on drone dataset in terms of accuracy, precision, recall, and F1 score with various machine learning and deep learning methods such as ML (Random forest (RF), Support Vector Machine (SVM), Decision Tree (DT), Linear Regression (LR), Logistic Regression (LR), Naive Bayes (NB), Multiple Regression Analysis (MPA), K-Nearest Neighbor (KNN) and Perceptron Network (PN), DL (Recurrent Neural Network (RNN), Gated Recurrent Unit(GRU), Long short-term memory (LSTM) and Bi-LSTM (Bidirectional Long short-term memory). Experimental results reveal that the deep learning method has shown significant results for detecting intrusion and drone attacks. It can be seen in Fig. 9 that linear regression; decision tree and random forest results are quite well as compared to naive Bayes and the rest of machine learning methods but comparatively low as deep Learning methods. Table 4 highlighted that LSTM and Bi-LSTM accuracy is better than GRU and RNN. The RNN shows the lowest result in terms of accuracy, precision, recall, and f1 score.

Table 5 shows experimental results based on deep learning methods RNN, GRU, LSTM, and Bi-LSTM with different iterations (number of epochs) and Decay. It has been reported that the number of epochs is one significant parameter for the training and testing of the model. When the model is trained on a few epochs, the model's accuracy is compromised, and the error ratio is relatively high. When we increased the iteration, the model gradually covered. Furthermore, there is no substantial difference between 30 to 50 epochs. The iterations model is based on dataset and resources; therefore, it is decided that a maximum of 100 epochs is adequate. Another hypermeter that influences the overfitting and underfitting if the ratio of the neuron is low in each layer, the chance of the model to be underfitting with inaccurate simulation, and the model will also lose significant features in that case. If the ratio of neurons in the layer is high, the chances for overfitting and the model will only learn given features or limited features. The model uses a dropout and regularization approach to overcome such conditions, randomly deactivating several neurons.

**Table 5:** Performance Analysis of Machine Learning and Deep Learning (results of model gradually converged).

Method	Epoch	Decay	Lr	Run time	Train	Test
RNN	30	1 × 10^−3^	1 × 10^−4^	0:00:12	0.90	0.91
	50	1 × 10^−3^	1 × 10^−4^	0:00:50	0.91	0.90
	100	1 × 10^−3^	1 × 10^−4^	0:01:09	0.92	0.90
GRU	30	1 × 10^−4^	1 × 10^−2^	0:00:11	0.86	0.83
	50	1 × 10^−2^	1 × 10^−2^	0:00:55	0.89	0.87
	100	1 × 10^−4^	1 × 10^−3^	0:01:05	0.89	0.87
LSTM	30	1 × 10^−4^	1 × 10^−3^	0:00:19	0.87	0.82
	50	1 × 10^−4^	1 × 10^−3^	0:00:49	0.87	0.82
	100	1 × 10^−4^	1 × 10^−2^	0:01:09	0.90	0.82
Bi-LSTM	30	1 × 10^−4^	1 × 10^−3^	0:00:08	0.90	0.86
	50	1 × 10^−4^	1 × 10^−2^	0:00:54	0.89	0.86
	100	1 × 10^−4^	1 × 10^−2^	0:01:11	0.91	0.88

Moreover, during the detailed analysis of the model's performance, we computed the learning rate (LR) and the Decay of the model as presented in Table 3. The decay calculates the model's learning rate (LR) in each iteration (epoch). It shows how much learning is down on iteration. Table 3 also shows various evaluation criteria for comparing RNN, GRU, LSTM, and Bi-LSTM. It also highlighted that each method has the best result on 100 epochs. Comparatively, the model's performance in the testing stage (1–3%) is lower than in the training stage. As per the result summary with different methods in Table 3, the model Bi-LSTM and simple RNN method perform well on 100 epochs. The training and testing accuracy of RNN and LSTM (91%, 91%), respectively. The LSTM network has long-term memory, which stores information with the help of the forget gate. It specifies how much previous memory is kept. Each iteration of the LSTM network returned backwards and updated weights with biases.

In Table 6, each row represents an attack type, and the columns display the precision, recall, and F1 score values corresponding to that attack type. Please note that the values in this table are hypothetical examples and should be replaced with the actual results obtained from the LSTM model trained on the CSE-CIC-IDS 2018 dataset. The accuracy or correctness of identifying and classifying brute force attacks is relatively better than other attacks on the dataset. It measures the proportion of true positive predictions (correctly identified attacks) out of the total predicted positive instances (all instances identified as attacks).

**Table 6:** Precision, Recall, and F1 Score on LSTM.

Attack type	Precision	Recall	F1 score
Botnet attack	0.92	0.85	0.88
HTTP DoS	0.78	0.82	0.80
Web app attacks	0.91	0.93	0.92
Network infiltration	0.83	0.77	0.80
Brute force	0.94	0.92	0.93
DDoS	0.87	0.88	0.87

## Conclusion

This paper proposed an IoT-Empowered smart cyber security framework called the Internet of Drones (IoDs), a drone-based network using machine learning and deep learning methods. This proposed framework uses IoT-based data from sensors, sensors network, and drone-enabling devices information to achieve security level patterns in identifying security threats and exploiting attack patterns. Also, we presented a holistic view of the drones/UAVs and provided a detailed explanation and classification of IoT Empowered smart cyber security networks. The proposed framework has been reported to be effective for detecting cyberattacks on challenging datasets. The proposed framework achieved outstanding results with deep learning methods (RNN and LSTM), which is comparatively better than traditional ML methods. In addition, the precision, recall, and F1-score are computed for detailed analysis to estimate the performance. The presented framework reveals generalizability and robustness for identifying attack types. Finally, imputable to alarmingly increase and use of drones in terrorism and crime, further studies will be conducted to prevent and counter the UAV threats.

## Data Availability

The datasets used/analysed during the current study are available at the following links: https://www.kaggle.com/datasets/cicdataset/cicids2017, https://www.kaggle.com/datasets/galaxyh/kdd-cup-1999-data.

## References

[1] Diaz Linares, I.; Pardo, A.; Patch, E.; Dehghantanha, A.; Choo, K.K.R. IoT Privacy, Security and Forensics Challenges: An Unmanned Aerial Vehicle (UAV) Case Study. In Handbook of Big Data Analytics and Forensics; Springer: Berlin, Germany, 2022; pp. 7–39.

[2] Aloqaily M, Boucher O, Boukerche A, Al Ridhawi I (2021). Design guidelines for blockchain-assisted 5G-UAV networks. IEEE Netw..

[3] Aldhyani TH, Alkahtani H (2022). Attacks to automatous vehicles: A deep learning algorithm for cybersecurity. Sensors.

[4] Aloqaily M, Hussain R, Khalaf D, Hani D, Oracevic A (2022). On the role of futuristic technologies in securing UAV-supported autonomous vehicles. IEEE Consum. Electron. Mag..

[5] Abdani, S.R.; Zulkifley, M.A.; Zulkifley, N.H. A lightweight deep learning model for covid-19 detection. In Proceedings of the 2020 IEEE Symposium on Industrial Electronics & Applications (ISIEA), Kuala Lumpur, Malaysia, 17–18 July 2020; pp. 1–5.

[6] Gharibi M, Boutaba R, Waslander SL (2016). Internet of drones. IEEE Access.

[7] Khan AA, Wagan AA, Laghari AA, Gilal AR, Aziz IA, Talpur BA (2022). BIoMT: A state-of-the-art consortium serverless network architecture for healthcare system using blockchain smart contracts.". IEEE Access.

[8] Grieco, L.A.; Boggia, G.; Piro, G.; Jararweh, Y.; Campolo, C. Ad-Hoc, Mobile, and Wireless Networks. In Proceedings of the 19th International Conference on Ad-Hoc Networks and Wireless, ADHOC-NOW 2020, Bari, Italy, 19–21 October 2020; Springer Nature: Berlin, Germany, 2020; Volume 12338.

[9] Rademacher T (2020). Artificial Intelligence and Law Enforcement.

[10] A. Muhammad, M. Asad, and A. R. Javed, “Robust early stage botnet detection using machine learning,” in 2020 International Conference on Cyber Warfare and Security (ICCWS). IEEE, 2020, pp. 1–6.

[11] Saif WS, Esmail MA, Ragheb AM, Alshawi TA, Alshebeili SA (2020). Machine learning techniques for optical performance monitoring and modulation format identification: A survey. IEEE Commun. Surv. Tutor..

[12] Ala'a Al-Habashna. "Building Height Estimation using Street-View Images, Deep-Learning, Contour Processing, and Geospatial Data." *CRV*. 2021.

[13] Barletta VS, Caivano D, Nannavecchia A, Scalera M (2019). A spell checking web service API for smart city communication platforms. Open J. Appl. Sci..

[14] Chang C-W, Lee H-W, Liu C-H (2018). A review of artificial intelligence algorithms used for smart machine tools. Inventions.

[15] Charan, DL Rama, et al. "Convolutional Neural Network based Water Resource Monitoring Using Satellite Images." 2020 5th International Conference on Communication and Electronics Systems (ICCES). IEEE, 2020.

[16] Estrada, Elsa. "Smart City visualization tool for the Open Data georeferenced analysis utilizing machine learning." Instituto de Ciencias Sociales y Administración (2018).

[17] Fedorova, Stanislava. GANs for Urban Design. Preprint at https://arXiv.org/quant-ph/2105.01727 (2021).

[18] Moosavi, V. "Urban morphology meets deep learning: Exploring urban forms in one million cities, towns and villages across the planet. arXiv e-prints, page. Preprint at https://arXiv.org/quant-ph/1709.02939 (2017).

[19] Supramongkonset, J.; Duangsuwan, S.; Promwong, S. A WiFi Link Budget Analysis of Drone-based Communication and IoT Ground Sensors. In Proceedings of the 2021 7th International Conference on Engineering, Applied Sciences and Technology (ICEAST), Pattaya, Thailand, 1–3 April 2021; pp. 234–237.

[20] Yin, Z.; Song, Q.; Han, G.; Zhu, M. Unmanned optical warning system for drones. In Global Intelligence Industry Conference (GIIC 2018); International Society for Optics and Photonics: Bellingham, DC, USA, 2018; Volume 10835, p. 108350Q.

[21] Koslowski R, Schulzke M (2018). Drones along borders: Border security UAVs in the United States and the European Union. Int. Stud. Perspect..

[22] Ozmen, M.O.; Yavuz, A.A. Dronecrypt-an efficient cryptographic framework for small aerial drones. In Proceedings of the MILCOM 2018–2018 IEEE Military Communications Conference (MILCOM), Los Angeles, CA, USA, 29–31 October 2018; pp. 1–6.

[23] Khan MA, Ullah I, Alsharif MH, Alghtani AH, Aly AA, Chen CM (2022). An efficient certificate-based aggregate signature scheme for internet of drones. Secure. Commun. Netw..

[24] Ranjitha, K.; Pathak, D.; Tammana, P.; Antony, F.A.; Alladi, T. Accelerating PUF-based UAV Authentication Protocols Using Programmable Switch. In Proceedings of the 2022 14th International Conference on COMmunication Systems & Networks (COMSNETS), Bangalore, India, 4–8 January 2022; pp. 309–313.

[25] Li S, Bai Y (2022). Deep learning and improved HMM training algorithm and its analysis in facial expression recognition of sports athletes. Comput. Intell. Neurosci..

[26] Tuli EA, Golam M, Kim DS, Lee JM (2022). Performance enhancement of optimized link state routing protocol by parameter configuration for UANET. Drones.

[27] Khan MA, Ullah I, Alkhalifah A, Rehman SU, Shah JA, Uddin II, Alsharif MH, Algarni F (2021). A provable and privacy-preserving authentication scheme for UAV-enabled intelligent transportation systems. IEEE Trans. Ind. Inform..

[28] Maghazei, O.; Netland, T.H.; Frauenberger, D.; Thalmann, T. Automatic drones for factory inspection: The role of virtual simulation. In Proceedings of the IFIP International Conference on Advances in Production Management Systems; Springer: Berlin, Germany, 2021; pp. 457–464.

[29] Kapoutsis AC, Michailidis IT, Boutalis Y, Kosmatopoulos EB (2021). Building synergetic consensus for dynamic gas-plume tracking applications using UAV platforms. Comput. Electr. Eng..

[30] Nguyen, H.P.D.; Nguyen, D.D. Drone application in smart cities: The general overview of security vulnerabilities and countermeasures for data communication. In Development and Future of Internet of Drones (IoD): Insights, Trends and Road Ahead; Springer: Berlin, Germany, 2021; pp. 185–210.

[31] Kumar A, Elsersy M, Darwish A, Hassanien AE, Kumar A, Elsersy M, Darwish A, Hassanien AE (2021). Drones combat COVID-19 epidemic: Innovating and monitoring approach. Digital Transformation and Emerging Technologies for Fighting COVID-19 Pandemic: Innovative Approaches.

[32] Aydin, Y.; Kurt, G.K.; Ozdemir, E.; Yanikomeroglu, H. Group authentication for drone swarms. In Proceedings of the 2021 IEEE International Conference on Wireless for Space and Extreme Environments (WiSEE), Cleveland, OH, USA, 12–14 October 2021; pp. 72–77.

[33] Saha HN, Roy R, Chakraborty M, Sarkar C, Choudhury A, Biswas A, Prateek M, Chakrabarti A (2021). IoT-enabled agricultural system application, challenges and security issues. Agricultural Informatics: Automation Using the IoT and Machine Learning.

[34] Liu C, Zhan R (2022). Global analysis of active defense technologies for unmanned aerial vehicle. IEEE Aerosp. Electron. Syst. Mag..

[35] Jares, G.A.; Valasek, J. Flight Demonstration and Validation of Control Acquisition Autopilot Attack. In Proceedings of the AIAA SciTech 2022 Forum, San Diego, CA, USA, 3–7 January 2022; p. 2341.

[36] Talaei Khoei T, Ismail S, Kaabouch N (2022). Dynamic selection techniques for detecting GPS spoofing attacks on UAVs. Sensors.

[37] Kafi MA, Challal Y, Djenouri D, Doudou M, Bouabdallah A, Badache N (2013). A study of wireless sensor networks for urban traffic monitoring: Applications and frameworks. Procedia Comput. Sci..

[38] Mansfield, K.; Eveleigh, T.; Holzer, T.H.; Sarkani, S. Unmanned aerial vehicle smart device ground control station cyber security threat model. In Proceedings of the 2013 IEEE International Conference Technology Homel Security (HST), Waltham, MA, USA, 12–14 November 2013; pp. 722–728.

[39] Khan AA, Laghari AA, Shafiq M, Awan SA, Gu Z (2022). Vehicle to everything (V2X) and edge computing: A secure lifecycle for UAV-assisted vehicle network and offloading with blockchain. Drones.

[40] Eyerman J, Hinkle K, Letterman C, Schanzer D, Pitts W, Ladd K (2013). Unmanned Aircraft and the Human Element: Public Perceptions and First Responder Concerns; Institute of Homeland Security and Solutions.

[41] Khan, Abdullah Ayub, Asif Ali Laghari, Zaffar Ahmed Shaikh, Zdzislawa Dacko-Pikiewicz, and Sebastian Kot. "Internet of Things (IoT) security with blockchain technology: a state-of-the-art review." IEEE Access (2022).

[42] Rahman, M.F.B.A. Smart CCTVS for Secure Cities: Potentials and Challenges; Rajaratnam School of International Studies (RSIS): Singapore, 2017.

[43] Kim, A.; Wampler, B.; Goppert, J.; Hwang, I.; Aldridge, H. Cyber Attack Vulnerabilities Analysis for Unmanned Aerial Vehicles. Aerospace Res. Cent. 2012, 2438.

[44] Zeng Y, Zhang R, Lim TJ (2016). Wireless communications with unmanned aerial vehicles: Opportunities and challenges. IEEE Commun. Mag..

[45] Soria PR, Bevec R, Arrue BC, Ude A, Ollero A (2016). Extracting objects for aerial manipulation on UAVs using low-cost stereo sensors. Sensors.

[46] Erdelj, M.; Natalizio, E. Drones, Smartphones and Sensors to Face Natural Disasters. In Proceedings of the 4th ACM Workshop on Micro Aerial Vehicle Networks, Systems, and Applications, Paris, France, 10–15 June 2018; pp. 75–86.

[47] Son, Y.; Shin, H.; Kim, D.; Park, Y.; Noh, J.; Choi, K. Rocking Drones with Intentional Sound Noise on Gyroscopic Sensors. In Proceedings of the 24th USENIX Security Symposium, Washington, DC, USA, 12–14 August 2015.

[48] Zhi Y, Fu Z, Sun X, Yu J (2019). Security and privacy issues of UAV: A survey. Mob. Netw. Appl..

[49] Strohmeier M, Schafer M, Lenders V, Martinovic I (2014). Realities and challenges of nextgen air traffic management: The case of ADS-B. IEEE Commun. Mag..

[50] Hooper, M.; Tian, Y.; Zhou, R.; Cao, B.; Lauf, A.P.; Watkins, L.; Robinson, W.H.; Alexis, W. Securing commercial WiFi-based UAVs from common security attacks. In Proceedings of the MILCOM 2016–2016 IEEE Military Communications Conference, Baltimore, MD, USA, 1–3 November 2016; pp. 1213–1218.

[51] Hartmann, K.; Giles, K. UAV exploitation: A new domain for cyber power. In Proceedings of the 2016 8th International Conference Cyber Conflict, Tallinn, Estonia, 31 May–3 June 2016; pp. 205–221.

[52] Rivera, E.; Baykov, R.; Gu, G. A Study on Unmanned Vehicles and Cyber Security. In Proceedings of the Rivera 2014 ASO, Austin, TX, USA, 2014.

[53] Junejo IN, Foroosh H (2010). GPS coordinates estimation and camera calibration from solar shadows. Comput. Vis. Image Underst..

[54] Shakhatreh H, Sawalmeh AH, Al-Fuqaha A, Dou Z, Almaita E, Khalil I, Othman NS, Khreishah A, Guizani M (2019). Unmanned aerial vehicles (UAVs): A survey on civil applications and key research challenges. IEEE Access.

[55] Cook, K.L.B. The Silent Force Multiplier: The History and Role of UAVs in Warfare. In Proceedings of the 2007 IEEE Aerospace Conference, Big Sky, MT, USA, 3–10 March 2007; pp. 1–7.

[56] Siddiqi, M.A.; Khoso, A.M. Aziz, Analysis on Security Methods of Wireless Sensor Network (WSN). In Proceedings of the SJCMS 2018, Sukkur, Pakistan, 10 December 2018.

[57] Kong W, Li X, Hou L, Yuan J, Gao Y, Yu S (2022). A reliable and efficient task offloading strategy based on multi-feedback trust mechanism for IoT edge computing. IEEE Internet Things J..

[58] Pushpa SX, Raja SK (2022). Elliptic curve cryptography based authentication protocol enabled with optimized neural network based DoS mitigation. Wirel. Pers. Commun..

[59] Sengan S, Khalaf OI, Sharma DK, Hamad AA (2022). Secured and privacy-based IDS for healthcare systems on E-medical data using machine learning approach. Int. J. Reliab. Qual. Healthc. (IJRQEH).

[60] Shaikh ZA, Khan AA, Teng L, Wagan AA, Laghari AA (2022). BIoMT modular infrastructure: The recent challenges, issues, and limitations in blockchain hyperledger-Enabled E-healthcare application. Wirel. Commun. Mobile Comput..

[61] Shaikh ZA, Khan AA, Baitenova L, Zambinova G, Yegina N, Ivolgina N, Laghari AA, Barykin SE (2022). Blockchain hyperledger with non-linear machine learning: A novel and secure educational accreditation registration and distributed ledger preservation architecture. Appl. Sci..

[62] Aldaej A, Ahanger TA, Atiquzzaman M, Ullah I, Yousufudin M (2022). Smart cybersecurity framework for IoT-empowered drones: Machine learning perspective. Sensors.

[63] Qureshi KN, Rana SS, Ahmed A, Jeon G (2020). A novel and secure attacks detection framework for smart cities industrial internet of things. Sustain. Cities Soc..

[64] Khan AA, Shaikh AA, Shaikh ZA, Laghari AA (2022). Karim S IPM-Model: AI and metaheuristic-enabled face recognition using image partial matching for multimedia forensics investigation with genetic algorithm. Multim. Tools Appl..

[65] Muhammad AN, Aseere AM, Chiroma H, Shah H, Gital AY, Hashem IAT (2020). Deep learning application in smart cities: Recent development, taxonomy, challenges and research prospects. Neural Comput. Appl..

[66] Bhattacharya S, Somayaji SRK, Gadekallu TR, Alazab M, Maddikunta PKR (2020). A review on deep learning for future smart cities. Internet Technol. Lett..

[67] Elsaeidy AA, Jagannath N, Sanchis AG, Jamalipour A, Munasinghe KS (2020). Replay attack detection in smart cities using deep learning. IEEE Access.

[68] Singh SK, Jeong Y-S, Park JH (2020). A deep learning-based IoT oriented infrastructure for secure smart city. Sustain. Cities Soc..

[69] Chen D, Wawrzynski P, Lv Z (2020). Cyber security in smart cities: A review of deep learning-based applications and case studies. Sustain. Cities Soc..

[70] Vinayakumar R, Alazab M, Srinivasan S, Pham Q-V, Padannayil SK, Simran K (2020). A visualized botnet detection system based deep learning for the internet of things networks of smart cities. IEEE Trans. Ind. Appl..

[71] Ferrag MA, Maglaras L, Moschoyiannis S, Janicke H (2020). Deep learning for cyber security intrusion detection: Approaches, datasets, and comparative study. J. Inform. Secur. Appl..

[72] Magaia N, Fonseca R, Muhammad K, Segundo AHFN, Neto AVL, de Albuquerque VHC (2020). Industrial Internet of things security enhanced with deep learning approaches for smart cities. IEEE Internet Things J..

[73] Javed AR, Usman M, Rehman SU, Khan MU, Haghighi MS (2020). Anomaly detection in automated vehicles using multistage attention-based convolutional neural network. IEEE Trans. Intell. Transp. Syst..

[74] Afzal S, Asim M, Javed AR, Beg MO, Baker T (2021). Urldeepdetect: A deep learning approach for detecting malicious urls using semantic vector models. J. Netw. Syst. Manag..

[75] Andresen G, Appice A, Di Mauro N, Loglisci C, Malerba D (2020). Multi-channel deep feature learning for intrusion detection. IEEE Access.

[76] Khan AA, Laghari AA, Shafiq M, Cheikhrouhou O, Alhakami W, Hamam H, Shaikh ZA (2022). Healthcare ledger management: A blockchain and machine learning-enabled novel and secure architecture for medical industry. Hum.-Centric Comput. Inform. Sci..

[77] Manzoor I, Kumar N (2017). A feature-reduced intrusion detection system using ANN classifier. Expert Syst. Appl..

[78] Kou L, Ding S, Ting Wu, Dong W, Yin Y (2022). An intrusion detection model for drone communication network in SDN environment. Drones.

[79] Musafer H, Abuzneid A, Faezipour M, Mahmood A (2020). An enhanced design of sparse autoencoder for latent features extraction based on trigonometric simplexes for network intrusion detection systems. Electronics.

[80] Ramadan RA, Emara A-H, Al-Sarem M, Elhamahmy M (2021). Internet of drones intrusion detection using deep learning. Electronics.

[81] Khan AA, Laghari AA, Gadekallu TR, Shaikh ZA, Javed AR, Rashid M, Estrela VV, Mikhaylov A (2022). A drone-based data management and optimization using metaheuristic algorithms and blockchain smart contracts in a secure fog environment. Comput. Electr. Eng..

[82] Yao H, Fu D, Zhang P, Li M, Liu Y (2018). MSML: A novel multilevel semi-supervised machine learning framework for intrusion detection system. IEEE Internet Things J..

[83] Jia Y, Wang M, Wang Y (2019). Network intrusion detection algorithm based on deep neural network. IET Inf. Secur..

